# Rapid quantification of bioactive compounds in *Salvia miltiorrhiza Bunge* derived decoction pieces, dripping pill, injection, and tablets by polarity-switching UPLC-MS/MS

**DOI:** 10.3389/fchem.2022.964744

**Published:** 2022-07-15

**Authors:** Qing Shen, Haixing Wang, Bin Quan, Xiuhua Sun, Guohua Wu, Darong Huang, Qingcheng Wang, Pei Luo

**Affiliations:** ^1^ State Key Laboratories for Quality Research in Chinese Medicines, Faculty of Pharmacy, Macau University of Science and Technology, Hangzhou, China; ^2^ Zhejiang Province Joint Key Laboratory of Aquatic Products Processing, Collaborative Innovation Center of Seafood Deep Processing, Institute of Seafood, Zhejiang Gongshang University, Hangzhou, China; ^3^ Zhejiang Province Key Lab of Anesthesiology, The Second Affiliated Hospital and Yuying Children’s Hospital of Wenzhou Medical University, Wenzhou, China; ^4^ Hangzhou Linping Hospital of Traditional Chinese Medicine, Hangzhou, China; ^5^ Zhejiang Provincial People’s Hospital, Affiliated People’s Hospital of Hangzhou Medical College, Hangzhou, China

**Keywords:** salvia miltiorrhiza bunge, ultra performance liquid chromatography, polarity switching, tandem mass spectrometry, rapid quantification

## Abstract

Salvia miltiorrhiza Bunge (SMB) has unambiguous biological functions in cardiovascular diseases, thus has been processed into different medicine forms. However, universal analytical method for fast quantification of bioactive compounds in SMB and SMB derived products is still missing. In this study, a polarity switching strategy was developed and optimized, which enabled the detection of the target compound in both positive and negative ion modes in a single run. The MS2 features of each compound were characterized to select the most prominent transitions for quantitative and qualitative analysis. Afterwards, the performance of this method was validated in terms of linearity (≥0.9916), limit of detection (LOD, 0.003–0.135 ngml^−1^), limit of quantification (LOQ, 0.010–0.450 ngml^−1^), precision (48.23 ± 2.58 ngml^−1^ to 53.72 ± 3.11 ngml^−1^), recovery (RSD 2.04%–5.79%), and stability (RSD ≤7.52%). Finally, the bioactive compounds in SMB and SMB derived products were determined, among which salvianate A, salvianolic acid A, and rosmarinic acid were the main components in all samples. In conclusion, the polarity switching UPLC-MS/MS method is efficient in accurate determining the bioactive compounds, which greatly shorten the time for analysis when compared with conventional methods. It has great potential quality control of SMB and SMB derived products.

## Introduction

Salvia miltiorrhiza (S. miltiorrhiza, SM) is one of the most commonly used traditional Chinese medicine, originated from the dry root and rhizome of Salvia miltiorrhiza Bunge plant in Lamiaceae family. It is widely distributed in both China and Japan, playing the roles of important health resource and potential economic resource (Zhang et al., 2010). Recently, SMB has been gradually acknowledged as a health product in western countries due to its significant and reliable biological activities. Nowadays, SMB is officially registered in the Chinese Pharmacopoeia (2020), as well as in US Pharmacopoeia 36 as a dietary supplement (Gu et al., 2006).

The remarkable pharmacodynamics and wide use of SMB have attracted researchers’ attention. It is reported that SMB ameliorates atherogenesis in the aortas of HFD-fed db/db mice *via* inhibiting KLF10 expression and upregulating HO-1, subsequently reducing ROS generation in VSMCs (Zhou et al., 2020). The aqueous extract of SMB Radix Puerariae herb pair significantly improved vascular injury in diabetic mouse model possibly through decreasing oxidative stress derived from NOX4 (Zhao et al., 2019). SMB also has a protective effect on estrogen-deficient bone loss manifested in ovariectomized and naturally menopaused mouse models, which seems to be associated with modulating signaling molecules involved in the progression of bone loss such as RANKL, OPG, and BALP, as well as the expression of genes involved in osteoclast activation such as cathepsin K, TRAF6, and NFATc1 (Lee et al., 2020). In clinical practice, SMB and its components are mainly used to treat cardiovascular diseases, including hypertension, diabetes, atherosclerosis, and chronic heart failure (Li et al., 2018).

Due to the unambiguous biological function, SMB has been processed into different forms, such as decoction pieces, dripping pill, injection, and tablets to enhance performance and make it easy taking (Jia et al., 2018). However, after the treatment of different processing technology, the contents of bioactive ingredients, such as diterpenoid quinones, hydrophilic phenolic acids and essential oil, which are the material basis for therapeutic role and the major prescribed pharmacodynamic substances, in those SMB derived products become unclear (Cao et al., 2020). By far, many methods have been developed to separate and analyze this kind of compounds in herbal medicines (Li et al., 2017; Zhang et al., 2018). For instance, Mei et al. (2021). determined 17 bioactive components in the fibrous roots of SMB by a combinatorial ultra performance liquid chromatography Ultraviolet (UPLC UV) characteristic spectra analysis. Lin et al. (2019). developed a protocol for the determination of bioactive compounds in the different organs of SMB by ultrahigh performance liquid chromatography-tandem mass spectrometry (UHPLC-MS/MS). Liu et al. (2007). Separated and determined three water-soluble components in SMB using flow injection-capillary electrophoresis system. Liu et al. (2019). employed nuclear magnetic resonance (NMR) based metabolomics and transcriptomics techniques to explore the biosynthesis mechanism of phenolic acid in SMB. However, these methods are carried out in a single ionization mode, while the active compounds in SMB are sensitive in different modes of MS/MS. For instance, salvianate A, rosmarinic acid, and salvianolic acid are sensitive in negative ion mode, while cryptotanshinone, dihydrotanshinone, tanshinone IIA, and tanshinone I are sensitive in positive ion mode, leading to that these compounds can’t be simultaneously detected in a single running. Besides, these methods are specific to the raw material of SMB, while a universal method for simultaneous determining bioactive compounds in decoction pieces, dripping pill, injection, and tablets of SMB is still lacking.

The aim of this study was to established a robust and efficient method on the basis of polarity switching UPLC-MS/MS technology for determining the main bioactive compounds in SMB and SMB derived products. The active compounds in SMB, no matter sensitive in positive- or negative ion modes, could be detected in a single running. This method was validated in terms of linearity, sensitivity, precision, and recovery, and could be implemented for quality control.

## Materials and methods

### Chemicals and reagents

Tanshinone I, tanshinone IIA, cryptotanshinone, dihydrotanshinone, salvianic acid A sodium, salvianolic acid A, and rosmarinic acid with purity ≥98% were purchased from Aladdin Bio-Chem Technology Co., Ltd. (Shanghai, China). HPLC grade solvents and reagents were all obtained from Merck (Darmstadt, Germany). Ultra-pure water (18.2 MΩ) was produced in-house by a Milli-Q water purification system (Millipore, France).

### Sample preparation and procedure

The primary stock solutions of 0.1 mgmL^−1^ of tanshinone I, tanshinone IIA, cryptotanshinone, dihydrotanshinone, salvianic acid A sodium, salvianolic acid A, and rosmarinic acid were prepared in methanol. A series of working standard solutions were prepared by diluting aliquots of primary solution with methanol. All solutions were stored at 4°C in glass tubes prior to use.

The samples of compound danshen dropping pill, thrombosis xinmaining tablet, Salvia miltiorrhiza Bunge, danshen injection, compound danshen tablet, and shexiang tongxin dripping pill were provided by Hangzhou Linping Hospital of Traditional Chinese Medicine (Hangzhou, Zhejiang).

The SMB derived decoction pieces, dripping pill, and tablets were ground into powder. There was 0.5 g powder accurately weighed and dissolved in 50 ml methanol in the help of ultrasonication for 10 min. Afterwards, the mixture was centrifuged at 4°C and 9,000 g for 10 min 1 ml of the supernate was picked and further diluted 10 times for analysis. Regarding danshen injection, 1 ml of the sample was diluted 1,000 times with Milli-Q water to make a sample solution for analysis. All of the solutions were filtered through a 0.22 μm membrane before analysis.

### Ultra performance liquid chromatography separation

Liquid chromatographic experiments were carried out on a Waters Acquity UPLC system (Waters, Milford, MA) including autosampler, quaternary pump, and vacuum degasser. After the clean-up and enrichment step, the analytes were injected onto a reversed-phase Waters HSS T3 C18 column (1.8 μm, 100 mm × 2.1 mm). The mobile phase (MP) was composed of MP-A the mixture of water (0.1% formic acid) and MP-B acetonitrile and be used under the gradient: the percent of MP-B was linearly changed from 10% to 90% in 5 min; 5–10 min, the percent of MP-B was kept constant at 90% for 5 min; 10–12 min, the percent of MP-B was decreased in 2 min. The flow rate was 0.3 mlmin^−1^ throughout the run time. Under these conditions, the chromatograms of the standards and samples were recorded.

### Mass spectrometry

Mass spectrometric analysis was carried out on a 5500QTrap system (Applied Biosystems, CA). The samples were ionized through an eletrospray ionization (ESI) ion source under positive/negative switching mode, which could switch polarities in 50 ms. Nitrogen was used as gas power, with the curtain gas 30 psi, GS1 50 psi, and GS2 50 psi. The IS of positive and negative ion modes were 5,500 and −4,500 eV, respectively. The most influential voltage parameters, declustering potential (DP) and entrance potential (EP), and MRM channels were summarized in Table 1. System control, spectra acquisition, and data analysis were performed by Analyst 1.6.3 (Applied Biosystems, CA).

**TABLE 1:** The MS and MS/MS conditions for the target compounds.

Name	Rt (min)	MRM channel	CE (eV)	DP (eV)
Cryptotanshinone	6.55	297.1>254.2*	34	108
		297.1>251.1	32	120
Dihydrotanshinone	5.98	278.9>233.2*	30	64
		278.9>261.2	25	60
Tanshinone IIA	7.19	295.2>249.1*	35	52
		295.2>277.3	28	89
Tanshinone I	6.51	277.2>249.1*	28	42
		277.2>193.1	35	52
Salvianate A sodium	2.23	196.7>134.9*	−22	−43
		196.7>122.9	−20	−48
Salvianolic acid A	3.76	493.1>294.9*	−21	−60
		493.1>184.7	−36	−50
Rosmarinic acid	3.55	359.0>196.9*	−21	−31
		359.0>160.9	−27	−22

### Statistic analysis

The identities of tanshinone I, tanshinone IIA, cryptotanshinone, dihydrotanshinone, salvianate A sodium, salvianolic acid A, and rosmarinic acid in SMB derived decoction pieces, injectoin, dripping pill, and tablets were confirmed by matching their retention times and MS2 data with corresponding standards. The analytical data was analyzed by one-way analysis of variance (ANOVA).

## Results and discussion

### Chromatographic behavior

**FIGURE 1:**
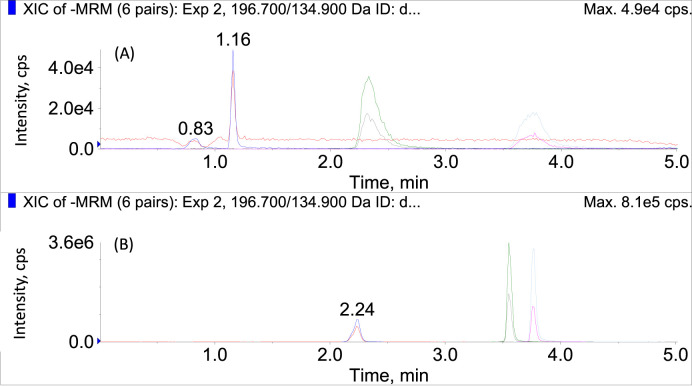
The chromatograms of salvianate A, rosmarinic acid, and salvianolic acid in MRM mode **(A)** before and **(B)** after the addition of formic acid in mobile phases.

Preliminary experiments indicated that the polarity switching mode could maintain an acceptable dwell time and number of points per peak, but did not influence the instrumental linearity or sensitivity adversely when compared to the conventional acquisition modes. Therefore, polarity switching mode was applied to analyze the bioactive compounds in SMB and SMB derived products within a single run. Acidic mobile phases (containing 0.1% formic acid) were preferred for the appreciable signal improvement for rosmarinic acid and salvianolic acid. Before acidification, the chromatographic peaks of these two compounds were flat and asymmetric with obvious tailing effect as displayed Figure 1A. After the addition of formic acid in mobile phases, although the peak shape of salvianate A sodium became worse more or less, both the peaks of rosmarinic acid and salvianolic acid became symmetric and sharp (Figure 1B). Therefore, 0.1% formic acid was used as the additive in mobile phases.

**FIGURE 2:**
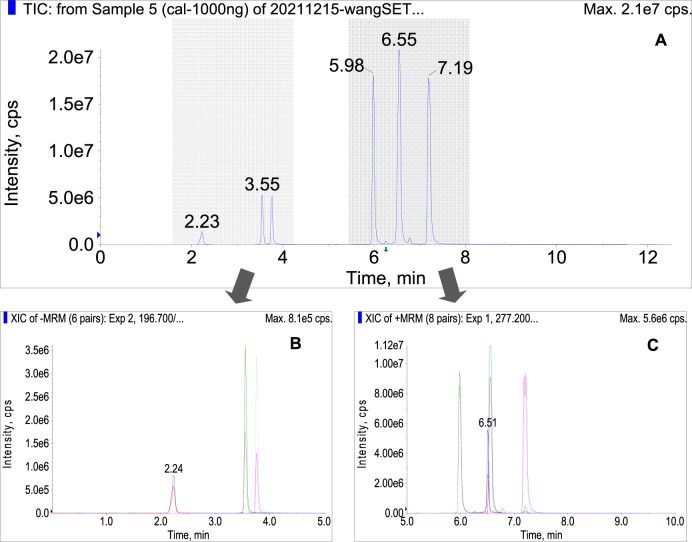
**(A)** total current chromatogram of the seven bioactive compounds under the optimized conditions in polarity switching mode, **(B)** MRM chromatogram of salvianate A, rosmarinic acid, and salvianolic acid in negative ion mode, **(C)** cryptotanshinone, dihydrotanshinone, tanshinone IIA, and tanshinone I in positive ion mode.

Under the optimized conditions, the target analytes were well separated in a single run within eight minutes (Figure 2A). They were eluted in the order of salvianate A sodium (2.23 min), rosmarinic acid (3.55 min), salvianolic acid A (3.76 min), dihydrotanshinone (5.98 min), tanshinone I (6.51 min), cryptotanshinone (6.55 min), and tanshinone IIA (7.19 min). The former three compounds were protonated in positive ion mode (Figure 2B), while the later four compounds were deprotonated in negative ion mode (Figure 2C). Almost all of the compounds of interest were separated either by chromatographic retention time or by SRM transition, except the tanshinone I and cryptotanshinone, which were overlapped and merged at 6.5 min in total ion current (TIC) chromatogram. Under MRM mode, these two compounds could be separated completely, because they owning different transitions, which will be explored in-depth in later section.

### MS characteristics of compounds

The fragmentation behavior of tanshinone I, tanshinone IIA, cryptotanshinone, dihydrotanshinone, salvianic acid A sodium, salvianolic acid A, and rosmarinic acid was investigated, to deeply understand the MS characteristics of these compounds.

**FIGURE 3:**
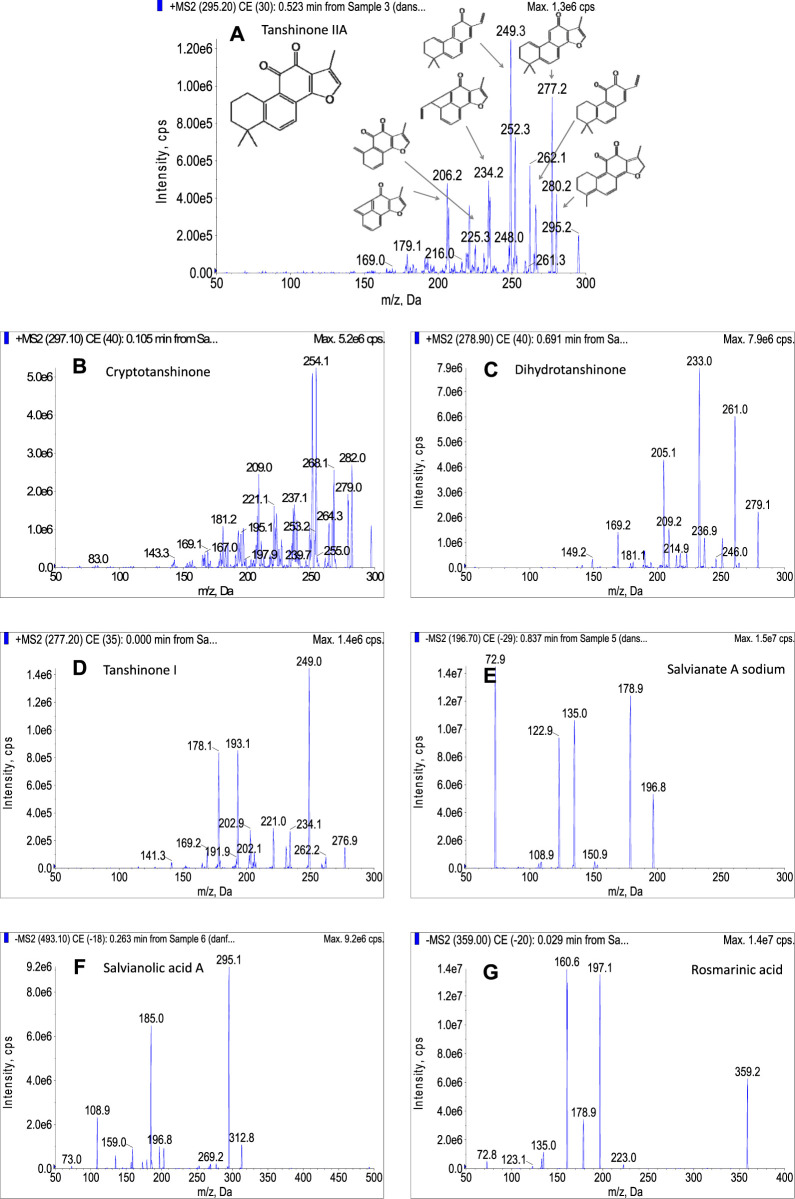
The secondary mass spectra of seven bioactive compounds.

The tanshinone IIA, cryptotanshinone, dihydrotanshinone, and tanshinone I were sensitive in positive ion mode. For tanshinone IIA, it was easily ionized in positive ion mode with proton adduction (m/z 295.2, [M + H]^+^). As shown in Figure 3A, the fragment ions m/z 280, 277, 266, 249, 235, 225, and other characteristic ions appeared in the secondary mass spectrogram after CID cracking. The fragment ions m/z 280 and 266 were speculated to be [M + H−CH_3_]^+^ and [M + H−CHO]^+^, mainly formed by losing a methyl group and aldehyde group, respectively. Tanshinone IIA is easy to lose a water molecule due to the existence of an unaromatic A ring, and the peak of [M + H−H_2_O]^+^ (m/z 277) is mainly displayed in the secondary spectrum, which was further degraded to the fragment ion m/z 249 by losing a −CO group. The fragment ions of m/z 235 and 207 were produced due to the rearrangement of the unaromatic A ring of the fragment m/z 277, the former (m/z 235) was formed by the loss of −C_3_H_6_− group, and the later was generated by the loss of −C_5_H_10_− group. This fragment pattern was changed when the CE was varied from 0 to 100 eV, and each ion has its own optimal condition for fragmentation. As a result, m/z 249 was found to be the most prominent ion, followed by m/z 277, and these two ions were used to pair with the parent ion as quantifier and qualifier transitions, respectively. For cryptotanshinone (Figure 3B), the structural characteristics of product ions were quite similar with those of tanshinone IIA. For instance, the product ions of m/z 254 [M−CH_3_−CO]^+^, m/z 251 [M−H_2_O−CO]^+^, m/z 282 [M−CH_3_]^−+^, m/z 279 [M−H_2_O]^+^, etc. were observed in the MS spectrum with significant intensities. For dihydrotanshinone (Figure 3C), the highest peak is m/z 233 [M−H_2_O−CO]^+^, followed by m/z 261 [M−H_2_O]^+^. As shown in Figure 3D, the main product ions of tanshinone I were generated resulting from the successive loss of −CO groups. It could be speculated that the fragment ion m/z 249 [M−CO]^+^ was formed by the loss of a molecule of −CO from the parent nucleus; the m/z 221 [M−2CO]^+^ was produced due to the loss of another molecule of −CO; the m/z 193 [M−3CO]^+^ was generated by further fragmenting a molecule of CO.

Under negative ion mode, the salvianate A sodium was taken as an example (Figure 3E), which was prone to be deprotonated as [M−H]^−^ as shown the peak of m/z 196.7 in the secondary mass spectrum. The peak of m/z 179 represented the fragment ion ([M−H−H_2_O]^−^) was produced from the loss of a water molecule from parent ion. A further loss of CO_2_ molecule occurred, resulting in the product ion of m/z 135 ([M−H−H_2_O−CO_2_]^−^). The ion of m/z 123 and 153 were speculated to be obtained by the loss of −CH_2_O_2_ group and CO2 molecule from the parent ion, respectively. For salvianolic acid A (Figure 3F), the two most intensive ions, m/z 295 and 185, were derived from the loss of caffeic acid moiety. The m/z 295 was speculated to be [M−caffeic acid−H_2_O]^−^ and the m/z 185 was [M−caffeic acid−CO]^−^. The negative parent ion for rosmarinic acid (Figure 3G) produced the fragments at m/z 161, 197, and 179. The fragment of m/z 197 was formed by the cleavage of the ester bond of rosmarinic acid and losing a caffeic acid moiety. An m/z ratio of 179 suggested the presence of caffeic acid moiety. The m/z 161 was obtained by subtracting a water molecule.

Based on the MS characteristics of the target compounds, the transitions used for MRM analysis were selected as shown in Table 1. For accurate quantifying each compound, at least 4 identification points are necessary (1 point is earned with the precursor ion and 1.5 points are earned with each product ions), thus two transitions between parent ion and the two most abundant product ions were used, one for quantitation and the other for identification.

### Performance validation

The performance of this well established polarity-switching UPLC-MS/MS method was validated according to Shen et al. (2010). (2010). The following performance studies were carried out: linearity, limit of detection (LOD) and limit of quantification (LOQ), precision, recovery and stability.

The experimental results were depicted in Table 2, the regression equations were linear in the ranges of 0.1–100 μg ml^−1^ for cryptotanshinone, dihydrotanshinone, tanshinone IIA, and tanshinone I and 0.5–1,000 μg ml^−1^ for salvianate A sodium, salvianolic acid A, and rosmarinic acid. The correlation coefficients (R2) were ≥0.9916, indicating a good linearity between the peak area (x) and the concentration of the target plasmalogens (y). LOD and LOQ are considered as the minimum concentrations of compound that can be identified and determined confidently by the proposed method. Under the optimized chromatographic conditions, the LOD and LOQ of each bioacitive compound in SMB derived medicines were calculated at a signal-to-noise-ratio (S/N) of three and ten, respectively. The LOD and LOQ values for the target compounds were 0.003–0.135 ngml^−1^ and 0.010–0.450 ngml^−1^, which indicated a high sensitivity of individual compound under the optimized chromatographic conditions. The precision was evaluated by testing the exact value of the spiked standard (50 ngml^−1^). The result showed that the mean value of each compound was varied in a small range from 48.23 ± 2.58 ngml^−1^ to 53.72 ± 3.11 ngml^−1^, indicating the good precision of this method. Recovery test was performed and the results were calculated on the basis of the equation: recovery (%) = 100 × (amount detected−original amount)/amount spiked (50 ngml^−1^). The mean recoveries expressed as relative standard deviation (RSD, %) were determined to be in the range of 2.04%–5.79%. The stability of the target compounds in samples was also investigated at the concentration level of 50 ngml^−1^ in triplicate. The test conditions included three storage periods at room temperature (24, 72 h, and 7 days). After being held at ambient temperature for the specified time, the target compounds were extracted and analyzed for a stability check. All the samples seem to be stable with RSD 7.52%.

**TABLE 2:** The results of method validation in terms of linearity, sensitivity, precision, and recovery.

Name	Linear range (ng·mL^−1^)	Equation	R2	LOD (ng·mL^−1^)	LOQ (ng·mL^−1^)	Precision^a^ (mean ± SD)	Recovery^a^ (RSD, %)
Cryptotanshinone	0.01–100	y = 1E-05x −1.5897	0.9919	0.009	0.030	48.23 ± 2.58	5.35
Dihydrotanshinone	0.01–100	y = 2E-05x −2.1578	0.9916	0.003	0.010	53.72 ± 3.11	5.79
Tanshinone IIA	0.01–100	y = 2E-05x −1.0208	0.9968	0.017	0.057	49.14 ± 2.12	4.31
Tanshinone I	0.01–100	y = 5E-05x −1.2928	0.9962	0.010	0.033	49.80 ± 1.73	3.47
Salvianate A sodium	0.05–100	y = 0.0003x −8.3986	0.9947	0.135	0.450	52.45 ± 2.08	3.97
Salvianolic acid A	0.05–100	y = 9E-05x + 0.1683	0.9981	0.125	0.417	50.08 ± 1.02	2.04
Rosmarinic acid	0.05–100	y = 0.0002x −8.1191	0.9954	0.090	0.300	47.58 ± 1.99	4.18

a Spiked level 50 ngmL^−1^.

### Bioactive compounds in salvia miltiorrhiza bunge derived medicines

**FIGURE 4:**
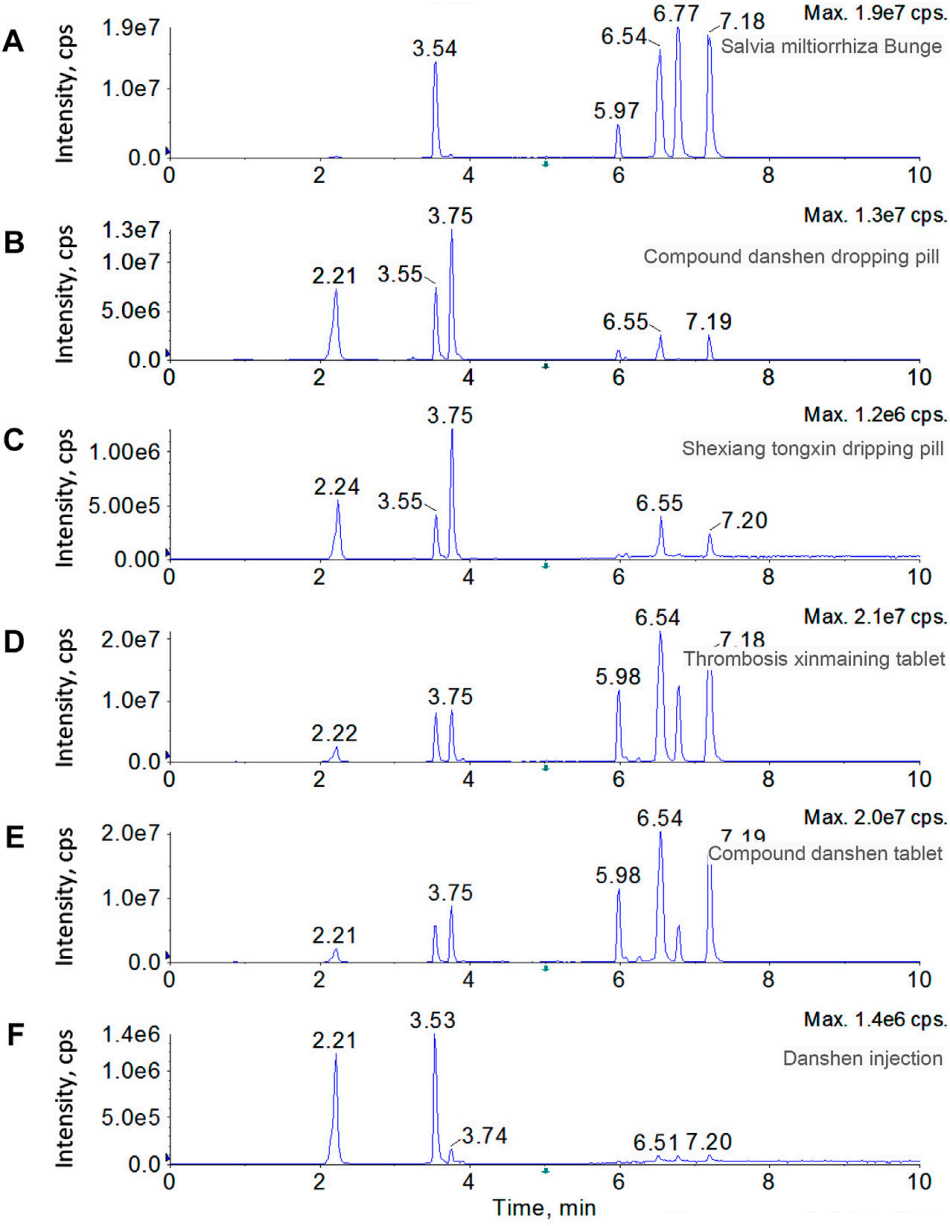
Chromatograms of SMB and five SMB derived products, including SMB decoction pieces, compound danshen dropping pill, thrombosis xinmaining tablet, danshen injection, compound danshen tablet, and shexiang tongxin dripping pill.

Because of the unambiguous biological functions in cardiovascular diseases, including hypertension, diabetes, atherosclerosis, and chronic heart failure, SMB has been processed into different forms, such as decoction pieces, dripping pill, injection, and tablets to enhance performance and make it easy taking. In this study, the SMB decoction pieces and five SMB derived products, including compound danshen dropping pill, thrombosis xinmaining tablet, danshen injection, compound danshen tablet, and shexiang tongxin dripping pill, were prepared for analysis. The spectrum for each sample displayed totally distinct chromatographic patterns (Figure 4). Besides the target seven bioactive compounds, a dihydrotanshinone isomer was also observed in several samples, including SMB, thrombosis xinmaining tablet, and compound danshen tablet. The contents of the target compounds were determined through the standard curves, and the dihydrotanshinone isomer was determined by the equation of dihydrotanshinone. As results shown in Table 3, in SMB, rosmarinic acid (5,835.28 μg g^−1^) was found to be the most abundant compound, followed by tanshinone I (1,346.56 μg g^−1^) and tanshinone IIA (999.16 μg g^−1^). The dihydrotanshinone isomer was calculated to be 856.92 μg g^−1^. For dripping pills, although the compound danshen dropping pill and shexiang tongxin dripping pill had similar chromatographic profile, they had significant difference in absolute contents. In compound danshen dropping pill, salvianate A sodium (6,998.10 μg g^−1^) took the high proportion among the target analytes, followed by rosmarinic acid (2,729.28 μg g^−1^) and salvianolic acid A (2,278.34 μg g^−1^). The contents of these three compounds were almost 100 times higher than those of cryptotanshinone, dihydrotanshinone, tanshinone IIA, and tanshinone I. In shexiang tongxin dripping pill, salvianate A sodium (418.26 μg g^−1^) was also the most abundant. Dihydrotanshinone and dihydrotanshinone isomer were not detected in shexiang tongxin dripping pill. For tablets, thrombosis xinmaining tablet and compound danshen tablet behaved almost identical chromatogram. Both these two medicines had the highest contents of rosmarinic acid (2,780.68 μg g^−1^ and 2017.88 μg g^−1^), salvianate A sodium (2025.18 μg g^−1^ and 1800.54 μg g^−1^), and salvianolic acid A (1,516.40 μg g^−1^ and 1,411.64 μg g^−1^). The major difference between these two medicines is the content of tanshinone I, which was rich in thrombosis xinmaining tablet (887.21 μg g^−1^) but rare in compound danshen tablet (97.24 μg g^−1^). In danshen injection, salvianate A sodium (897.72 μg g^−1^) and rosmarinic acid (518.70 μg g^−1^) were detected to be the dominant components, while the rest analytes were neglectable.

**TABLE 3:** The contents of the seven bioactive compounds in SMB and SMB derived products.

Name	Compound danshen dropping pill (μg·g^−1^)	Thrombosis xinmaining tablet (μg·g^−1^)	Salvia miltiorrhiza Bunge (μg·g^−1^)	Danshen injection (μg·mL^−1^)	Compound danshen tablet (μg·g^−1^)	Shexiang tongxin dripping pill (μg·g^−1^)
Cryptotanshinone	36.38	481.33	230.19	—	431.30	4.31
Dihydrotanshinone	25.81	408.80	162.28	—	392.54	—
Tanshinone IIA	98.01	973.74	999.16	5.37	918.16	10.47
tanshinone I	66.53	887.21	1,346.56	8.67	97.24	11.74
Salvianate sodium	6,998.10	2025.18	193.19	897.72	1800.54	418.26
Salvianolic acid A	2,278.34	1,516.40	62.73	40.60	1,411.64	214.49
Rosmarinic acid	2,729.28	2,780.68	5,835.28	518.70	2017.88	135.40
Dihydrotanshinone isomer	0.88	428.48	856.92	1.22	180.50	—

As a summary, the target compounds were more or less detected to be positive in the tested SMB and SMB derived medicines. The medicines in the same form but with different brands have generally the same pattern of the bioactive compounds. This phenomenon could be probably attributed to the similar medicine processing technology. The compounds in injection are relative rare, due to the complex purification steps. In addition, the proposed polarity switching UPLC-MS/MS method is robust and efficient in accurate determining the main bioactive compounds in SMB and SMB derived products.

## Conclusion

A rapid and efficient UPLC-MS/MS method was developed and validated for the analysis of main bioacitve compounds in SMB and SMB derived products. Rapid polarity switching in the heated ESI source was implemented for simultaneous analysis of positive and negative ionized compounds in a single chromatographic run of 8 min. This method maximizes the ionization efficiency, minimizes potential interference from co-eluted compounds, and provides a high S/N ratio in MRM chromatograms of the target compounds. The method was successfully applied to provide the chromatographic patterns of SMB derived dripping pills, tablets and injection. Overall, this work demonstrates the high analytical capabilities of the polarity switching UPLC-MS/MS method and provides the first insight into the potential of this platform for sensitive determination of bioactive compounds in SMB and SMB derived products.

## Data Availability

The original contributions presented in the study are included in the article/Supplementary Material, further inquiries can be directed to the corresponding authors.
